# Sub‐Nanogram Resolution Measurement of Inertial Mass and Density Using Magnetic‐Field‐Guided Bubble Microthruster

**DOI:** 10.1002/advs.202403867

**Published:** 2024-05-22

**Authors:** Leilei Wang, Minjia Sheng, Li Chen, Fengchang Yang, Chenlu Li, Hangyu Li, Pengcheng Nie, Xinxin Lv, Zheng Guo, Jialing Cao, Xiaohuan Wang, Long Li, Anthony L. Hu, Dongshi Guan, Jing Du, Haihang Cui, Xu Zheng

**Affiliations:** ^1^ State Key Laboratory of Nonlinear Mechanics Beijing Key Laboratory of Engineered Construction and Mechanobiology Institute of Mechanics Chinese Academy of Sciences Beijing 100190 China; ^2^ School of Building Services Science and Engineering Xi'an University of Architecture and Technology Xi'an 710055 China; ^3^ School of Engineering Science University of Chinese Academy of Sciences Beijing 100049 China; ^4^ Key Laboratory for Biomechanics and Mechanobiology of Ministry of Education Beijing Advanced Innovation Center for Biomedical Engineering School of Biological Science and Medical Engineering Beihang University Beijing 100083 China; ^5^ The High School Affiliated to Renmin University of China Beijing 100080 China

**Keywords:** bubble microthruster, hydrodynamic jet flow, magnetic manipulation, mass density of embryo, sub‐nanogram resolution

## Abstract

Artificial micro/nanomotors using active particles hold vast potential in applications such as drug delivery and microfabrication. However, upgrading them to micro/nanorobots capable of performing precise tasks with sophisticated functions remains challenging. Bubble microthruster (BMT) is introduced, a variation of the bubble‐driven microrobot, which focuses the energy from a collapsing microbubble to create an inertial impact on nearby target microparticles. Utilizing ultra‐high‐speed imaging, the microparticle mass and density is determined with sub‐nanogram resolution based on the relaxation time characterizing the microparticle's transient response. Master curves of the BMT method are shown to be dependent on the viscosity of the solution. The BMT, controlled by a gamepad with magnetic‐field guidance, precisely manipulates target microparticles, including bioparticles. Validation involves measuring the polystyrene microparticle mass and hollow glass microsphere density, and assessing the mouse embryo mass densities. The BMT technique presents a promising chip‐free, real‐time, highly maneuverable strategy that integrates bubble microrobot‐based manipulation with precise bioparticle mass and density detection, which can facilitate microscale bioparticle characterizations such as embryo growth monitoring.

## Graphical abstract

This work demonstrates a substantial progress of using swimming microrobots to perform precise tasks with sophisticated functions. This magnetic‐field‐guided bubble microthruster technique presents a promising chip‐free, real‐time, highly maneuverable strategy that integrates bubble microrobot‐based manipulation with precise bioparticle mass and density detection with sub‐nanogram resolution. This technique can facilitate microscale bioparticle characterizations such as embryo growth monitoring.

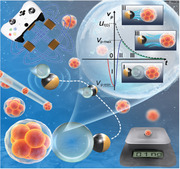

## 1 Introduction

The emerging technique of artificial micro/nano‐motors^[^ ^1^, ^2^, ^3^, ^4^, ^5^, ^6^, ^7^, ^8^ ^]^ provides a perfect example of employing miniature machines to accomplish tasks in the microscopic realm, as envisioned by Richard Feynman in his renowned speech “There is plenty of room at the bottom”.^[^ ^9^ ^]^ These micro/nano‐motors can harness energy from the ambient environment through chemical reactions^[^ ^10^, ^11^, ^12^ ^]^ or external fields such as acoustic,^[^ ^13^, ^14^ ^]^ magnetic,^[^ ^15^, ^16^, ^17^, ^18^, ^19^, ^20^, ^21^ ^]^ and light,^[^ ^22^, ^23^ ^]^ exhibiting autonomous movements. With integration of manipulation strategies based on hydrodynamics and external field steering, micro/nano‐machines or micro/nano‐robots have recently been developed for various applications, including targeted drug delivery,^[^ ^24^, ^25^, ^26^, ^27^, ^28^ ^]^ minimally invasive surgery,^[^ ^29^ ^]^ and microrobotic manipulation.^[^ ^30^, ^31^ ^]^

Among the various micro/nano‐motors, the microbubble‐driven micromotor is unique as it is capable of achieving the highest propulsion speed.^[^ ^10^, ^32^ ^]^ This is attributed to the high surface energy of the bubble and the focused hydrodynamic jet during bubble collapse, which significantly enhances the micromotor's propulsion. Contrary to the earlier concepts that relied on manipulating an external field and modifying the micromotor's geometry, structure, or surface,^[^ ^33^, ^34^, ^35^, ^36^ ^]^ the microbubble performs a variety of functions for the micromotor based on bubble dynamics and induced hydrodynamic flow,^[^ ^10^, ^37^, ^38^ ^]^ rather than solely providing energy. For instance, a bubble microrobot has been developed to fulfill multimodal functions like gripping, pushing, and anchoring, demonstrating high maneuverability and easy switching between different speeds and higher degrees of freedom.^[^ ^10^, ^39^ ^]^ The bubble microrobot has been applied as a key tool in a microfabrication platform at the air–liquid interface for 2D soft functionalized films or microelectronics.^[^ ^6^, ^17^ ^]^

Microbubbles introduce a wealth of additional functionalities through their intricate mechanisms. Specifically, the collapse of microbubbles and the resulting hydrodynamic impact generate a transient inertial effect, which holds significant value in the microscopic domain. Analogous to the scallop theorem,^[^ ^40^ ^]^ this inertial effect is crucial for disrupting time‐reversal reciprocal motion in viscous, low Reynolds number *Re* (*Re* is defined as a ratio of inertial effect to viscous effect) flows, essential for designing artificial microswimmers.^[^ ^11^, ^41^, ^42^ ^]^ Moreover, this inertial effect presents an opportunity to measure physical quantities at the microscale, which are closely tied to inertia but challenging to quantify using existing methods. For example, measuring the mass and mass variation of a bioparticle, such as an embryo or living cell, with a size of ≈10 µm could yield quantitative insights into cell growth, death, compositional changes, and responses to drug treatments.^[^ ^43^, ^44^, ^45^ ^]^ Achieving a mass detection resolution of 0.1 ng is necessary for microparticles of this size, while mass detection of ≈1 ng is challenging for traditional measurement techniques. Compared to previous methods relying on nano resonators^[^ ^45^, ^46^, ^47^, ^48^, ^49^, ^50^, ^51^ ^]^ or Raman scattering microscopy,^[^ ^44^ ^]^ bubble micromotors or microrobots offer an alternative approach for probing the mass or density of tiny particles through their response to inertial impacts. This approach boosts advantages such as controllable single‐particle selection, easy manipulation, and real‐time measurements.

Therefore, this study presents a novel approach employing a controllable bubble microthruster (BMT) to measure the inertial mass and density of tiny particles with sub‐nanogram resolution (0.1 ng = 10^−10^ g, which is ≈3% of the mass of a hepatocyte). The BMT comprises a Janus microsphere (JM) with a ferromagnetic nickel (Ni) layer embedded on its surface, which serves as a microbubble generator, and a microbubble, whose collapse generates an inertial “thrust” for both power and function. We establish a 3D Helmholtz electromagnetic coils (3D HEC) platform for facile BMT manipulation using a gamepad, and the exertion of inertial impact from the microbubble onto a target microparticle is facilitated through microscopic observation (refer to Experimental Section for details). The transient kinematic response of the target microparticle, recorded by an ultra‐high‐speed camera (with a frame rate of up to 450 000 fps), is analyzed based on our theoretical model. The inertial mass and density of the microparticle are determined by identifying the relaxation time *τ_p_*, which characterizes the transient movement of the microparticle. Subsequently, we elucidate the principle of the BMT and validate its efficacy and reliability based on results obtained for various microparticles with controlled sizes and densities. Furthermore, the BMT is employed to measure the mass densities of mice embryos, demonstrating its potential application in monitoring the density or mass variation of different bioparticles, such as embryo and cell growth. The platform for manipulating BMT and microparticles, HEC system, and image analysis software can be integrated into a programmable and multifunctional microrobotic system.

## 2 Principle

### 2.1 Principle of Inertial Mass/Density Measurement

**Figure 1:**
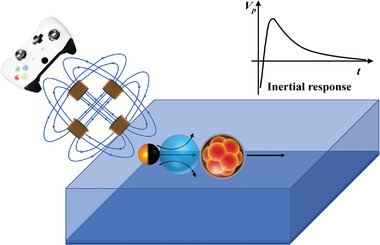
Schematic diagram of the magnetic‐field‐guided bubble microthruster (BMT), controllable via a gamepad. The BMT directs the energy of the collapsed microbubble to impact target microparticles, such as bioparticles, enabling measurement of their inertial mass and density based on their response to the impact.

**Figure 2:**
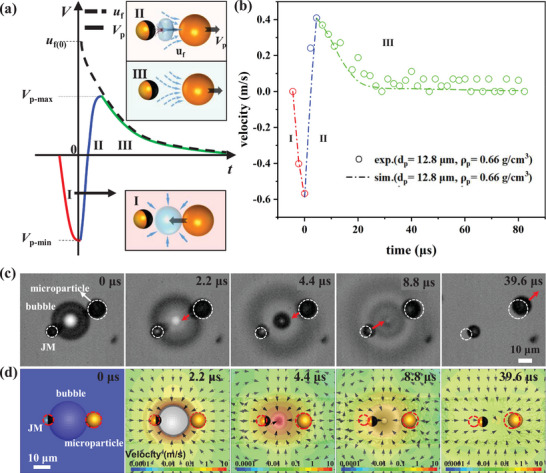
Illustration of the BMT principle. The principle relies on the transient velocity variation of the target microparticle in response to the inertial impact from the bubble collapse when a JM–bubble–particle configuration is established. a) Schematic diagram illustrating the three‐stage velocity variation *V_p_* of the target microparticle. In stage I (red), the microparticle retracts into the bubble cavity following the collapse. In stage II (blue), the transient hydrodynamic flow propels the microparticle strongly, resulting in a positive change in velocity. In stage III (green), the microparticle gradually decelerates as it interacts with the surrounding fluid flow. The dashed curve depicts the decay of the ambient fluid velocity *u_f_*. b) Measured velocity variation during stages II and III of a microparticle (with radius *R_p_* = 6.4 µm, density *ρ_p_* = 0.66 g cm^−3^) impacted by the BMT, compared with the dashed curve obtained from numerical simulation, indicating good agreement. c) Experimental snapshots (bottom view from the inverted microscope, see SM Video S1 (Supporting Information), recorded by an ultra‐high‐speed camera at 450 000 fps) capturing a BMT during bubble collapse, with white circles denoting the initial position of the target microparticle. d) Snapshots from numerical simulation showing the flow field and the motion of the microparticle at the same times as in (c). The red dashed circles display the original positions of the JM and the microparticle. The simulation perfectly reproduces the motions of both JM and the microparticle in experiment shown in (c).

In the experiment, we utilized a gamepad to maneuver the BMT toward the target microparticle (**Figure** **1**; SM Video S1, Supporting Information). The BMT's translational motion is driven by catalysis on the platinum surface of the Janus micromotor (JM), while its orientation and steering are controlled by a magnetic field generated by a 3D HEC system. Upon approach, the JM–bubble–particle configuration is established by positioning the bubble in the middle. Hydrodynamic theory and simulations^[^ ^10^, ^37^ ^]^ have previously predicted a robust hydrodynamic jet flow resulting from the collapse of a bubble. The direction of this jet flow is influenced by the surrounding confinement: in the JM–bubble–particle configuration, the flow will be directed toward the side with stronger confinement, depending on the size. As depicted in **Figure** **2**a, the BMT operates effectively when the diameter of the JM is smaller than that of the target microparticle, termed as “pusher mode”,^[^ ^10^ ^]^ wherein the jet flow propels the microparticle away from its original position.

The transient response of the target microparticle propelled by the BMT is crucial for measuring its inertial mass/density and can be divided into three stages, as depicted in Figure 2a. In stage I (red curve), immediately after the bubble collapse, the microparticle retracts into the bubble cavity under the influence of the surrounding fluid that fills the cavity. In stage II (blue curve), a hydrodynamic jet flow toward the particle rapidly forms after the bubble cavity is filled with fluid, causing an instantaneous change in the particle's velocity *V_p_* from negative to positive. Stage III commences when the particle's speed reaches its maximum value, equivalent to the fluid speed *u_f_* (shown as the dashed curve in Figure 2a). In stage III (green curve), the particle's speed *V_p_* gradually decreases in tandem with the decay of the surrounding fluid flow *u_f_* (*t*) due to viscous dissipation.

This three‐stage velocity variation is validated through our experiments using a microparticle with a radius of *R_p_* = 6.4 µm, observed by an ultra‐high‐speed camera at 450 000 fps, as illustrated in Figure 2b and the experimental snapshots in Figure 2c. Notably, stage II is a rapid process lasting ≈4.4 µs, while stage III takes a much longer time, exceeding 50 µs. It is worth mentioning that the duration of bubble collapse is known to be 0.5*τ_R_* ≈ 3.9 µs (where $\tau_{R}=\sqrt{\rho {R_{b}}^{3}/\gamma }\;$ = 7.8 µs is the inertial Rayleigh timescale, and the bubble radius is *R_b_* = 16.2 µm),^[^ ^52^, ^53^ ^]^ which closely aligns with the 4.4 µs duration observed in our experiment. Additionally, we conduct numerical simulations to visualize the transient flow field during bubble collapse (Figure 2d; see Figure S3 of Supporting Information for details), providing valuable insights for analyzing the hydrodynamic drag force exerted on the target particle. In the simulation, the diameters of the JM, bubble, and microparticle are 8.8, 32.4, and 12.8 µm, respectively. The simulation flow field corroborates the working principle described above, and the propelled displacement of the microparticle shown in Figure 2d within 40 µs is ≈7.6 µm, consistent with experimental observations. Furthermore, the *Re* number reaches ≈5 based on the maximum speed of the microparticle, which is ≈0.4 m s^−1^. This finite *Re* number clearly indicates the transient inertial effect introduced by the BMT during the aforementioned process.

Subsequently, we establish a kinematic equation for the microparticle's transient velocity variation *V_p_* (t) in stages II and III:

(1) $$m_{p}\;\frac{dV_{p}}{dt}=\;6\pi \mu R_{p}\left[u_{f}\left(t\right)-V_{p}\right]$$

where *m_p_* and *R_p_* denote the inertial mass and radius of the target microparticle, and *μ* is fluid viscosity. The relaxation time *τ_p_* = 2*R_p_* ^2^ *ρ_p_*/9*μ* is the crucial parameter in this kinematic equation, representing the characteristic time of the temporal variation of the inertial response *V_p_* (*t*). To address the challenge of solving the velocity *V_p_* (*t*) in Equation (1) due to the intricate flow field *u_f_* (*t*), we suggest an approach to model the temporal decay of *u_f_* (*t*). Based on our experimental observations, which reveal both rapid and slow decays of *u_f_* (*t*) in stage III, we propose an equation comprising two exponential components to describe the variation of the fluid velocity *u_f_* (*t*):

(2) $$u_{f}\left(t\right)=\left(c_{1}e^{-t/\tau 1}+c_{2}e^{-t/\tau 2}\right)$$

where *τ*_1_ and *τ*_2_ denote the typical timescales of the rapid and slow fluid velocity decay respectively in stage III, and the parameters c_1_ and c_2_ are determined by the initial velocity condition: *u_f_* (0) = c_1_ + c_2_. Using Equation (2) to represent the transient flow variation is validated by our numerical simulation, depicted by the dash‐dotted curve in Figure 2b. As discussed earlier, the rapid dynamics are characterized by the Rayleigh timescale *τ_R_*, where we observe *τ*_1_ = 0.5*τ_R_*. The slower decay follows a much longer timescale *τ*_2_, ≈100 µs, determined by the extended tail of the experimental data within the range of *t* = 30–100 µs. By substituting Equation (2) into Equation (1), we can fully solve the ordinary differential equation with the initial condition *V_p_* (0) obtained from the experimental observation of the largest negative velocity at *t* = 0:

(3) $$V_{p}\left(t\right)\;=e^{-t/\tau_{p}}\;\left[\frac{c_{1}\tau_{1}}{\tau_{1}-\tau_{p}}e^{\left(t/\tau_{p}-t/\tau_{1}\right)}+\frac{c_{2}\tau_{2}}{\tau_{2}-\tau_{p}}e^{\left(t/\tau_{p}-t/\tau_{2}\right)}\right]+Ce^{-t/\tau_{p}}$$

Here, the parameter *C* is closely related to the initial condition, that is, $C\;=V_{p}\;\left(0\right)-\left(\frac{c_{1}\tau_{1}}{\tau_{1}-\tau_{p}}+\frac{c_{2}\tau_{2}}{\tau_{2}-\tau_{p}}\right)$. Applying the experimental data in Equation (3), only two unknown parameters are obtained—the relaxation time of the target microparticle*τ_p_* and the initial fluid velocity *u_f_* (0). The microparticle's density *ρ_p_* is calculated as:

(4) $$\rho_{p}=9\mu t_{p}/2{R_{p}}^{2}$$

and the inertial mass is determined by multiplying the microparticle's volume by *ρ_p_*:

(5) $$m_{p}=6\pi \mu R_{p}\tau_{p}$$

### 2.2 Working Regime of BMT

The BMT operation relies on the establishment of the JM–bubble–particle configuration and subsequent hydrodynamic effects. It is also important to clarify the mechanism to direct the energy from the collapsing microbubble such that it impacts the target microparticle. In essence, the hydrodynamic mechanism is profoundly influenced by the asymmetric confinement imposed by both the JM and the target microparticle on either side of the bubble. Upon formation of a JM–bubble–particle assembly following gamepad manipulation, the collapse of the bubble induces a hydrodynamic jet flow directed toward the side of greater confinement.^[^ ^10^, ^37^ ^]^ If the target microparticle is larger than the JM, the hydrodynamic jet flow will be directed toward the microparticle, creating a “pusher” mode, referred to as the bubble microthruster or BMT in this study. Conversely, if the JM is larger than the target particle, a “puller” mode is established, resulting in the particle being drawn back toward the bubble. Clearly, the pusher mode is desirable for the BMT, while the puller mode should be avoided. It is worth noting that an “anchor” mode arises if the sizes of the JM and the particle are identical. Experimental videos illustrating these three modes are provided in SM Video S2 (Supporting Information).

**Figure 3:**
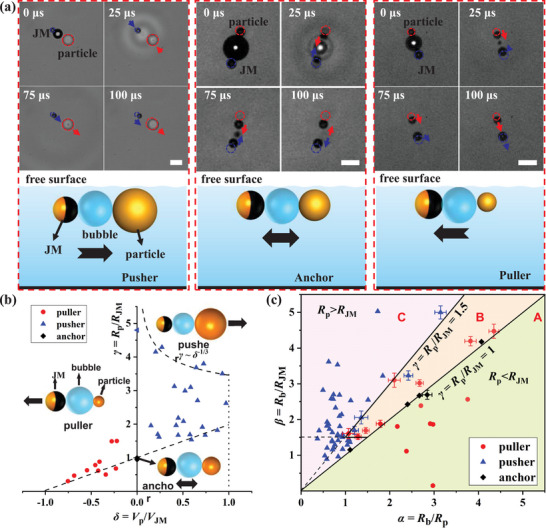
a) Representative snapshots and schematic diagram illustrating the three modes: pusher, anchor, and puller. The blue and red dotted circles indicate the initial positions of the JM and particle, respectively. Scale bar: 20 µm. Differentiating between the two fundamental working modes of the BMT: pusher versus puller. b) Phase diagram depicting the particle–JM size ratio *γ* versus the velocity ratio *δ*. c) Phase diagram showing the bubble–JM size ratio *β* versus the bubble–particle size ratio *α*. The experimental sample size *n* shown in (b) and (c) is up to *n* = 55.

Notably, the operational modes are contingent upon the size and velocity ratios between the JM and the target microparticle. **Figure** **3**a depicts representative snapshots and schematic diagrams of the various operational modes: pusher, puller, and anchor. We successfully establish phase diagrams presented in Figure 3b,c to demonstrate how to achieve the “pusher” mode for BMT operation, based on four dimensionless parameters: *α* = *R*_b_/*R_p_*, *β* = *R*_b_/*R*_JM_, *γ* = *R_p_*/*R*_JM_, and *δ* = $\bar{V_{p}}$/$\bar{V_{JM}}$, where *R*_b_, *R_p_*, and *R*_JM_ denote the radii of the bubble, the microparticle, and the JM, respectively. Here, $\bar{V_{p}}$ denotes the average velocity of the microparticle in a bubble cycle, while $\bar{V_{JM}}$ denotes the average velocity of the JM without loading the microparticle. Detailed mechanisms for distinguishing different modes are discussed later in Section 4. Briefly, for any target microparticle, we can specifically choose the size of the JM based on two key parameters *γ* and *β*, so that the BMT can work properly and efficiently. Based on the findings presented in Figure 3b,c, we can precisely control the BMT to operate in the “pusher” mode if the primary condition *γ* > 1.5 is met. To mitigate disturbance from large bubbles and ensure sufficient energy from small bubbles, we select 1 < *β* < 2.5 for the experiments shown below.

## 3 Results and Implementations

To validate the efficacy of the BMT, we initially employ polystyrene (PS) microspheres with various sizes and a fixed density of *ρ* = 1.05 g cm^−3^ as target microparticles. The measured sizes using our BMT method are in good agreement with values obtained through microscopic observation. Furthermore, we utilize hollow glass microspheres (HGMs) with adjustable densities to demonstrate the BMT's capability to tackle more challenging tasks. Additionally, we apply the BMT to measure the mass and density of different embryos, illustrating the feasibility of using the BMT as a real‐time, convenient, and high‐resolution method for probing mass/density variations of biological particles during various processes. The smallest JM utilized in our experiments was ≈*R*_JM_ = 3.4 µm, as bubble might not form on smaller JM surfaces due to a higher free energy barrier for bubble nucleation.^[^ ^54^ ^]^ Considering the threshold value of *γ* > 1.5, the smallest target microparticles are ≈*R_p_* = 5.1 µm, with a mass of ≈0.55 ng assuming a density of 1 g cm^−3^. Assuming a 20% measurement uncertainty, our method can achieve a mass resolution of ≈0.1 ng (which will be discussed later).

### 3.1 Verification Using PS Microspheres with Different Sizes

**Figure 4:**
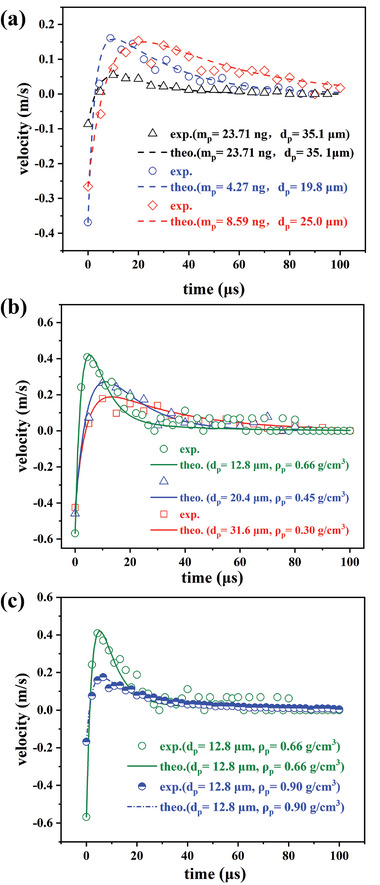
Comparison between the measured transient velocity variations and their fitted curves based on Equation (3). a) Results obtained using PS microspheres with fixed densities and different diameters *d_p_*. b) Results obtained using HGMs with varying diameters *d_p_* and effective densities *ρ_p_*. The inset displays the measured effective densities *ρ_p_* in relation to their diameters *d_p_*, consistent with the theoretical prediction of Equation (6) represented by the solid curve. c) The increase in effective density *ρ_p_* (blue dashed curve with half solid circles) of an HGM by coating a 40 nm platinum layer is accurately detected by BMT.

We first validated the BMT method using PS microspheres of varying sizes. Given the known density of PS microspheres (≈1.05 g cm^−3^), this measurement serves to confirm the accuracy of the BMT method. In **Figure** **4**a, the measured inertial masses of three distinct PS microspheres are 4.27, 8.59, and 23.71 ng, respectively, based on their measured diameters *d_p_* = 19.8, 25.0, and 35.1 µm, respectively, obtained from $d_{p}=\sqrt{18\mu \tau_{p}/\rho_{p}}$ fitting Equation (3). These PS microsphere diameters were also assessed using an optical microscope with a 100x/NA = 1.4 objective, yielding measured diameters of *d_p‐m_* = 20.4, 25.9, and 34.2 µm, respectively. The discrepancies are only 2.9%, 3.5%, and 2.6%, respectively, indicating a precision of ≈0.4 ng in measuring inertial mass. This result verifies the accuracy of the BMT method. Our experiments are typically conducted near the liquid–air surface due to the presence of bubbles. The theory based on Equation (3) may slightly underestimate the size and mass of the target microparticles because the microparticle could experience slightly lower drag force near the free liquid–air interface.

**Figure 5:**
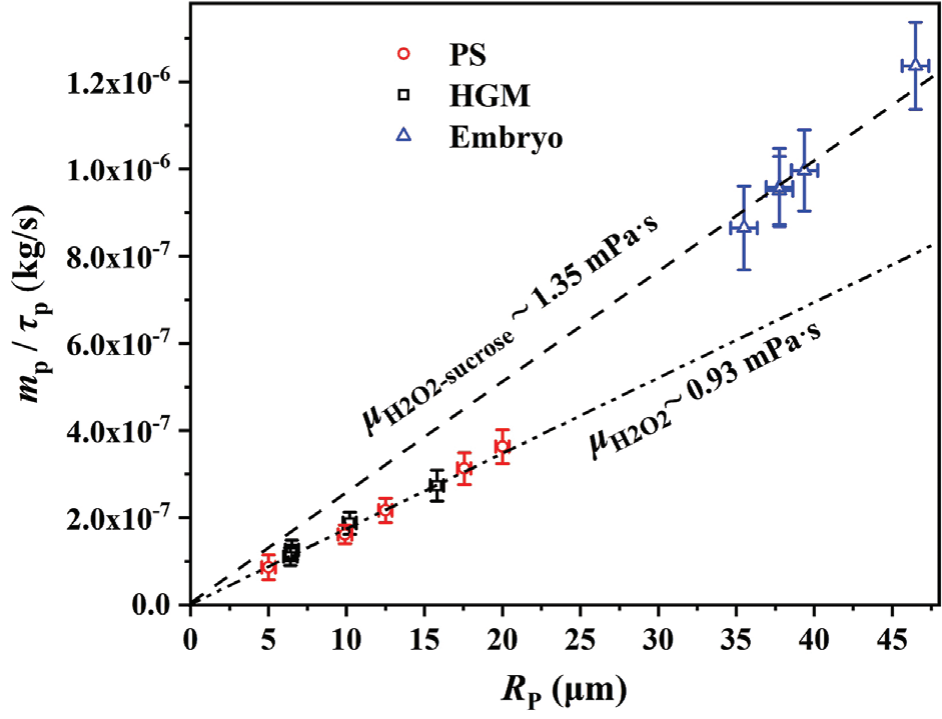
A comparison of all experimental data with the theoretical master curve *m_p_*/*τ_p_* versus *R_p_* based on Equation (5). The red circles are the results using PS microparticles discussed in Section 3.1, the black squares are the results of HGMs discussed in Section 3.2, and the blue triangles are the results of mice embryos discussed in Section 3.3. The measurement in the same solution follows the same master curve *m_p_*/*τ_p_* versus *R_p_* with the slope determined by the solution viscosity *μ*. PS microparticles and HGMs were measured in H_2_O_2_ solutions at ≈22 – 23 °C, manifesting a slope of *μ* = 0.93 mPa.s. The mice embryos were measured in 10% sucrose solutions whose viscosity is shown to be *μ* = 1.35 mPa.s. The error bars are evaluated based on 90% confidence interval of fitting.

Importantly, we notice that Equation (5) suggests a master curve of the BMT method, that is, *m_p_*/*τ_p_* = 6π*μR_p_*, which provides a more accurate assessment of the BMT method's measurement capabilities. As illustrated in **Figure** **5**, we plot the experimental data and compare them with the linear relation *m_p_*/*τ_p_* = 6π*μR_p_* derived from Equation (5). The red circles are the data using PS microparticles discussed above, and the dashed‐dotted line is plotted based on *m_p_*/*τ_p_* = 6π*μR_p_*, whose slope is only determined by the viscosity *μ* of H_2_O_2_ solution at ≈22 – 23 °C. Thus, the good linear tendency of the experimental data using the same solution in Figure 5 demonstrates robustness of the BMT method in detecting microparticle's mass with high precision.

### 3.2 Measuring Density of HGMs

Next, we demonstrated the capability of the BMT in determining the unknown density of a microparticle. HGMs with varying densities and made of different materials were chosen as examples of microparticles. The HGM comprises a glass shell (*ρ_shell_* = 2.23 g cm^−3^) and an internal air cavity. Consequently, the effective density of the HGMs decreases as their size increases. Initially, we utilized SEM to measure the shell thickness, yielding an average shell thickness of approximately *h* = 0.70 µm (see Figure S1, Supporting Information). Based on this result, we established a theoretical relationship between the effective density *ρ_p_* and the diameter *d_p_* of the HGM:

(6) $$\rho_{p}=\frac{\rho_{shell}}{{d_{p}}^{3}}\;\left[{d_{p}}^{3}-{\left(d_{p}-2h\right)}^{3}\right]+\frac{\rho_{air}}{{d_{p}}^{3}}{\left(d_{p}-2h\right)}^{3}$$

where *ρ_shell_* = 2.23 g cm^−3^ and *ρ_air_* = 0.00129 g cm^−3^ is the density of glass and air, respectively. The relationship described in Equation (6) is illustrated in Figure S2 and Table S1 (Supporting Information).

We employed the BMT method to measure the effective density of HGMs with various sizes, as depicted in Figure 4b. The fitting based on Equation (3) reveals that the effective density of an HGM with a diameter *d_p_* = 12.8 µm is approximately *ρ_p_* = 0.66 g cm^−3^, in close agreement with the predicted value of *ρ_p_* = 0.67 g cm^−3^ based on Equation (6). For larger HGMs (*d_p_* = 31.6 µm), the effective density decreases to approximately *ρ_p_* = 0.30 g /cm^−3^. In the inset of Figure 4b, we compare the measured data of effective density *ρ_p_* with the theoretical prediction curve from Equation (6). The excellent agreement underscores the effectiveness of the BMT method.

It is noteworthy that we can modify the density of the HGMs by coating them with a nanoscale platinum (Pt) layer of varying thickness *h*_Pt_ using E‐beam sputtering. The theoretical relation between the effective density *ρ_pt_* and the coating thickness *h*_Pt_ is:

(7) $$\rho_{p-pt}=\frac{\rho_{shell}}{{d_{p}}^{3}}\;\left[{d_{p}}^{3}-{\left(d_{p}-2h\right)}^{3}\right]+\frac{\rho_{air}}{{d_{p}}^{3}}{\left(d_{p}-2h\right)}^{3}+\frac{3\rho_{pt}}{d_{p}}h_{pt}$$

where *ρ_p‐pt_* = 21.45 g cm^−3^ is the density of Pt. For instance, by applying a Pt layer of *h*_Pt_ = 40 nm on one hemisphere of an HGM with a diameter ≈12.8 µm, the effective density of the HGM can be increased by ≈30% to *ρ_p‐pt_* = 0.86 g cm^−3^. The measured effective density of the coated HGM is approximately *ρ_p‐pt_* = 0.90 g cm^−3^ (Figure 4c), consistent with our prediction above (with a relative error of 4.6%). This demonstrates the high sensitivity of the BMT method in detecting the added mass of a nanoscale coating.

The measured results using HGMs are also shown in Figure 5 to demonstrate the robustness of the BMT method. As the same H_2_O_2_ solutions were used, the measured data using PS microparticles (red circles) and HGMs (black squares) all follows the same master curve that shows a viscosity of *μ* = 0.93 mPa.s. This viscosity is very close to the expected value *μ* = 0.95 mPa.s at 22 – 23 °C. The master curve can be applied to detect the mass and density of other microparticles.

### 3.3 Detection of Density Variation of Embryos

To showcase the versatility of the BMT method, we aimed to measure the density of embryos. A mouse embryo typically comprises an outer extraembryonic trophectoderm (TE) layer, an inner cell mass (ICM), and a fluid‐filled cavity,^[^ ^55^, ^56^ ^]^ and its density is expected to vary with cell division and cavity expansion during growth. This density variation could offer valuable insights into cell growth/division and composition changes, yet limited data are available. The embryos used were obtained from Institute of Cancer Research (ICR) female mice at the E3.75 – E4.0 stage. Before measurement, the embryos underwent washing in 1% (w/v) polyvinylpyrrolidone (PVP) (Sigma, PVP40) and fixation with 4% PFA (Sigma, P6148) for 30 min at room temperature. PFA (paraformaldehyde) is a common fixative used in cell biology and embryology to preserve cellular structures and prevent degradation. The fixation treatment facilitates further microscopic observation for morphology and detailed analysis of gene expression for the embryos collected from the BMT measurement, which makes the correlation of the embryo density variation with the embryo development possible.

**Figure 6:**
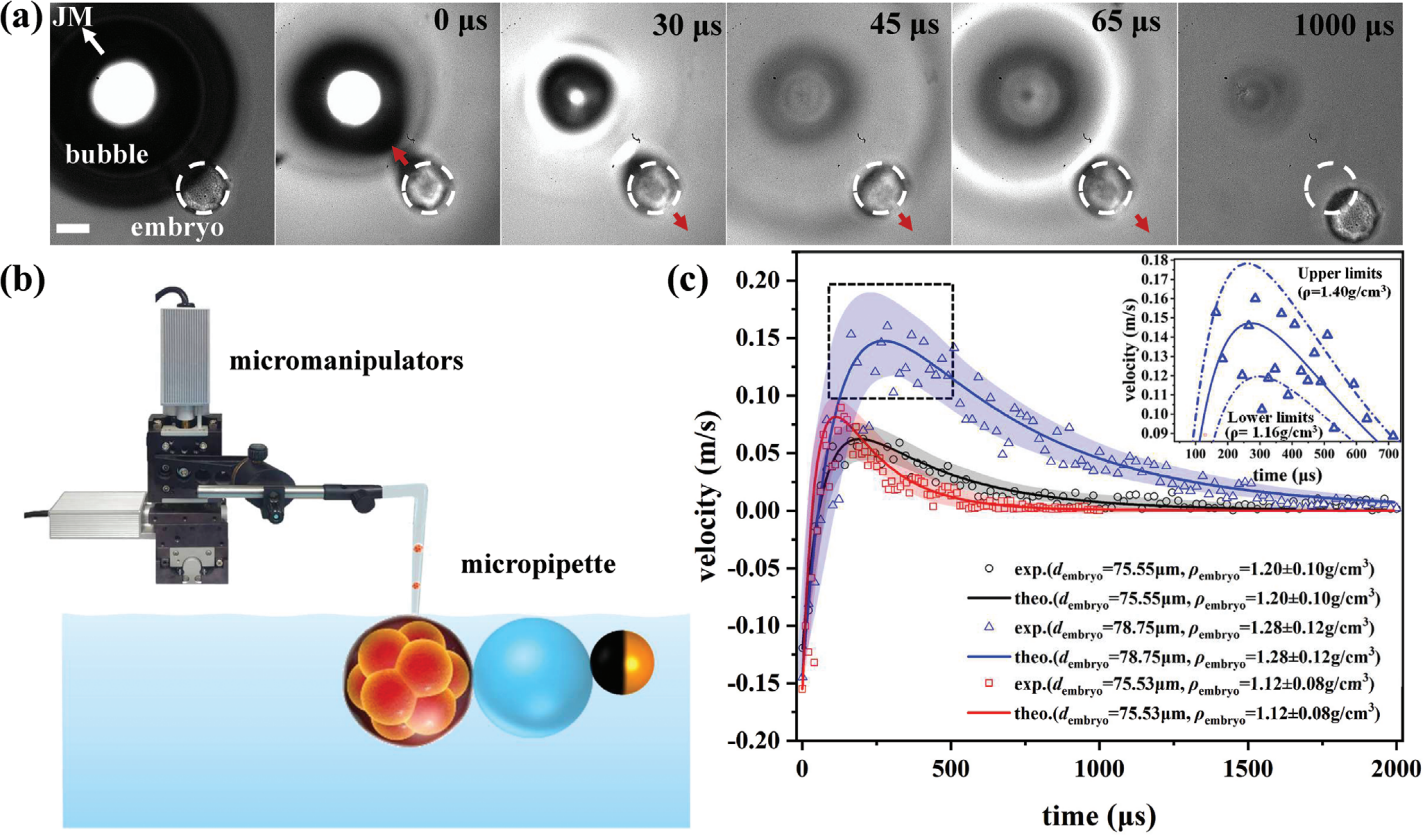
a) Experimental snapshots depicting the displacement of an embryo (diameter 78.75 µm) impacted by a BMT (with a bubble diameter ≈312.9 µm). The white dashed circle denotes the initial position of the embryo. Scale bar: 50 µm. b) Micropipette platform established to catch and drop the embryo near the BMT. c) Three measured curves of velocity variations of a single embryo. The solid curves represent the fit based on Equation (3). The colorful shadow region respectively displays the 90% confidence level of measurement uncertainty. Given that the embryo is much larger than the microparticle used above, the velocity peak resulting from the impact of the BMT is lower, while its width is broader.

To enable programmable manipulation and rapid detection, we devised a micropipette manipulation platform (**Figure** **6**b) to capture and position the embryo near the BMT. Given the non‐biofriendly nature of the current solution environment, this manipulation platform effectively reduces embryo contact time with the H_2_O_2_ solution.

For the BMT to function in the pusher mode, the embryo should be larger than the JM. In our experiments, the average embryo diameter is ≈75 µm (Figure 6a), while the JM diameter ranges from ≈35 to 40 µm. To ensure significant propulsion to the embryo, the microbubble diameter is increased to ≈312.9 µm. To prevent rapid embryo sedimentation after releasing the embryo from the micropipette, we added 10 wt.% sucrose to the solution to lower the density difference between the embryo and the solution.

The inertial response of the embryo to the impact of the BMT is depicted in Figure 6c (SM Video S3, Supporting Information). The measured data and the fit curve based on Equation (3) are presented in Figure 6c, revealing measured densities of 1.20 ± 0.10, 1.28 ± 0.12, and 1.12 ± 0.08 g cm^−^^3^, respectively. The measurement uncertainty is estimated at a 90% confidence level with its upper and lower boundaries shown by the colorful shadow region in Figure 6c (a zoom‐in of the peak region with the upper and lower boundaries is displayed in the inset of Figure 6c). From the average value of five independent measurements, we estimated that the density of mice embryos at the E3.75 – E4.0 stage (with diameters ranging from 72 – 93 µm) is ≈1.21 ± 0.12 g cm^−3^. These five data (blue triangles) and the master curve of *m_p_*/*τ_p_* versus *R_p_* are displayed in Figure 4b, showing robustness and consistence of the BMT method. The slope of the master curve (dashed line in Figure 5) corresponds to a viscosity *μ* = 1.35 mPa.s, which is close to the measured value *μ* = 1.45 mPa.s of the viscosity of 10% sucrose solution.

The experimental data indicate that the embryos in stage III exhibit a much longer decay time (≈1–2 ms) compared to the results shown in Figure 4 using PS and HGM microparticles. This prolonged decay is attributed to the larger size of the embryos, resulting in a significantly larger relaxation time *τ_p_* = 2*R_p_* ^2^ *ρ_p_*/9*μ* that varies with *R_p_* ^2^. It should be noted that the data points in Figure 6c appear more scattered compared to those in Figure 4, leading to a larger measurement uncertainty, ≈ ±10% of the measured value. From SM Video S3 (Supporting Information) and Figure 6a, we observe the influence of surface waves indicated by the propagation of a circular shadow, as both the BMT and the embryos are close to the air–liquid interface. These surface waves are stronger than in previous cases using smaller microbubbles (as shown in Figure 3c), resulting in more fluctuations in measuring embryo displacements. Nonetheless, the uncertainty stemming from scattered data can be mitigated by increasing the sampling number for statistical analysis.

## 4 Discussion

**Figure 7:**
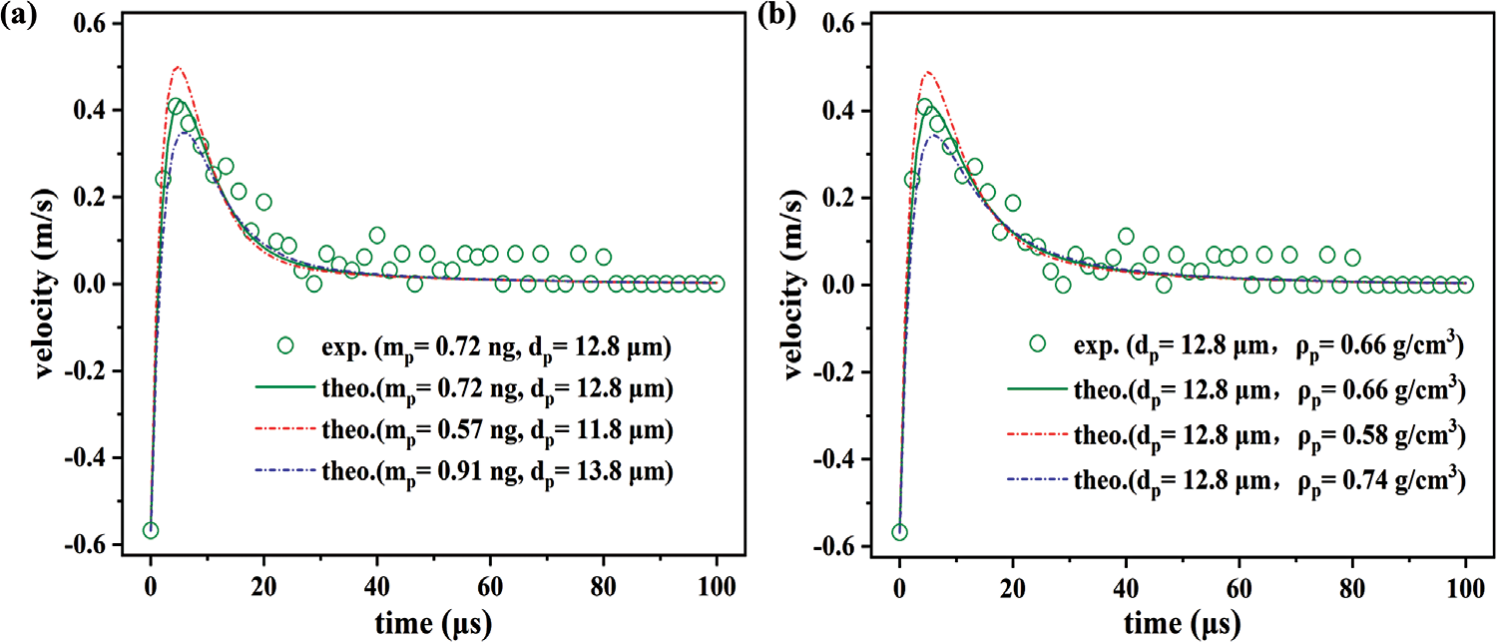
Measurement resolution of a) size and b) density.

In this section, we will discuss the extent to which the BMT technique can be employed. First, it is important to clarify the measurement resolution of the BMT method, which determines the smallest detectable mass of this technique. Using the example of an HGM (*d_p_* = 12.8 µm) with a measured mass *m*_p_ = 0.72 ng and *ρ*_p_ = 0.66 g cm^−3^, we illustrate the fit curve (green solid line) and the upper and lower boundaries of the fitting (red and blue dash‐dotted curves, respectively) in **Figure** **7**. Figure 7a demonstrates that the BMT method exhibits excellent size resolution, potentially smaller than *δd_p_* = 1.0 µm, with a relative deviation of <7.8%. This size resolution is equivalent to a high mass resolution of ≈0.15 – 0.18 ng, according to Equation (5). Additionally, Figure 7b shows that the BMT method provides an acceptable density resolution, with the measured result *ρ_p_* = 0.66 ± 0.08 g cm^−3^ indicating a resolution of 0.08 g cm^−3^ (relative deviation ≈12.1%). Notably, the resolution of the BMT method primarily depends on the positive peak of the measured velocity. Thus, for larger target microparticles, the resolution could be improved as the experiment collects more data in the peak region, resulting in a better fit. However, larger JM and bubble sizes are also necessary to enhance the peak velocity value.

We have mentioned in Section 2.2 that two dimensionless size parameters *γ* and *β* should be specifically determined so that the BMT can operate properly. The mechanism relies on hydrodynamic effect depending on the asymmetric confinement from both sides of the bubble. Figure 3b distinguishes the operational modes based on the microparticle–JM size ratio *γ* versus the velocity ratio *δ*. A value of *γ* = 1 indicates a symmetric system around the bubble, resulting in zero net displacement of the microparticle in a full bubble cycle. The reciprocal motions of both the microparticle and JM around the bubble characterize the “anchor” mode, with $\bar{V_{p}}$= 0 and *δ* = 0 (represented by a black diamond) in Figure 3b, as elucidated in our previous work.^[^ ^9^ ^]^ When *γ >* 1, the hydrodynamic jet flow directs toward the target particle, forming a “pusher” mode with *δ >* 0 (depicted as blue triangles in Figure 3b), necessary for the BMT function. Conversely, when 0 < *γ* < 1, the hydrodynamic jet flow is directed toward the JM, pulling the microparticle back to the JM side, resulting in a “puller” mode with *δ <* 0 (indicated by red circles in Figure 3b). When the microparticle's size is significantly smaller than that of the JM, the particle will completely follow the JM. In our experiments, we find that to achieve a stable pusher mode, the value of *γ* should exceed 1.5 rather than the ideal value of 1. Additionally, we delineate the upper and lower boundaries (dashed curves) of all the experimental data in the phase diagram in Figure 3b. The lower boundary approximately intersects (−1, 0) and (0, 1) in the *δ – γ* coordinates. The upper boundary follows a scaling of *γ* ∼*δ* ^−1/3^, dictated by the law of conservation of momentum (refer to Figure S2, Supporting Information).

Another phase diagram is constructed by considering the bubble effect, as illustrated in Figure 3c. The two axes are defined based on the bubble–JM size ratio *β* versus the bubble–particle size ratio *α*. We identify regime A, located in the lower right section where *R*_p_/*R*_JM_ = 1, representing a purely puller mode, and regime C, situated in the upper left section where *R*_p_/*R*_JM_ = 1.5, representing a purely pusher mode. An intermediate regime B falls within 1 < *R*_p_/*R*_JM_ < 1.5, where a stable pusher mode is attained when the bubble–JM size ratio is approximately *β* = *R*_b_/*R*_JM_ < 1.5. Beyond the threshold value of *β* = 1.5, the pusher mode transitions to a puller mode, attributed to the noticeable withdrawal of the microparticle into the bubble cavity if the bubble size is too large.

We plan to further measure the densities of different embryos and illustrate density variations during their growth. Additionally, it would be interesting to test the BMT method with various cell types such as HeLa, hepatocytes, and so on. However, without the protective TE layer of the embryo, these cells cannot survive for more than a few seconds in an H_2_O_2_ solution. To address this, we propose employing plasmonic microbubbles to replace the chemical decomposition reaction of the H_2_O_2_ solution.^[^ ^57^, ^58^ ^]^ The novel plasmonic method involves generating microbubbles by focusing a laser on an Au‐decorated particle in a bio‐compatible environment.

## 5 Conclusion

In summary, we demonstrated the effectiveness and accuracy of the BMT technique in quantifying the inertial mass and density of microparticles at sub‐nanogram resolution, which is a substantial advance of using bubble microrobot to perform precise tasks in microscopic world. The BMT harnesses energy from a collapsed microbubble to impart inertial impact on an adjacent microparticle through a hydrodynamic jet. The BMT was effortlessly controlled using a gamepad by integrating with magnetic field‐guided manipulation, which greatly enhanced its maneuverability. By measuring the transient velocity variation of the microparticle under such inertial impact and fitting it with an analytical solution from the ordinary differential kinematic equation, we determined the relaxation time *τ_p_*, and thereby obtained the mass and density of the microparticle. The master curves of the BMT method were also unveiled, which showed a linear dependence of *m_p_*/*τ_p_* on *R_p_*. We demonstrated the validity and effectiveness of the BMT method using PS microspheres and HGMs with different sizes and densities. The measured results indicate that the mass resolution could reach ≈0.1 ng, and the density resolution is ≈0.08 g cm^−3^. The BMT method was further applied to mice embryos, demonstrating its feasibility for use with biological microparticles. Distinct from previous methods that require complicated techniques like microcantilever resonator^[^ ^46^ ^]^ or stimulated Raman scattering microscopy,^[^ ^44^ ^]^ or that was limited to dry cells,^[^ ^45^ ^]^ our BMT method shows unique advantages by introducing a chip‐free, highly maneuverable, real‐time, and programable approach for sensing small masses and densities of bioparticles at high precision level. Particularly noteworthy is its potential application in monitoring density variations of embryos and cells, which could open new avenues for detecting cell growth/death, composition variation, and response to drug treatments.

## 6 Experimental Section

### Fabrication of Janus Microsphere

The fabrication of hollow magnetic Janus micromotors involved depositing Ni (≈40 nm) and Pt (≈20 nm) layers successively onto the hemispheres of hollow glass microspheres (HGMs). These HGMs, sourced from Sino‐steel Co. Ltd., ranged in diameter from ≈10 to 60 µm and were separated based on their particle size distributions using screening and flotation processes. During fabrication, a diluted suspension of HGMs was uniformly spread onto a hydrophilic polished silicon wafer and dried at 60 °C to create a monolayer of HGMs. Subsequently, Ni and Pt layers were sequentially deposited onto the surfaces of the HGMs using an electron beam evaporator. Due to the densely packed arrangement of the HGMs, the deposited Ni and Pt layers exclusively covered the upper hemispheres of the HGMs.

### Magnetic Manipulation System

A sophisticated magnetic actuation system was developed incorporating several key components for precise control. At its core were a computer, a user‐friendly gamepad for input, two signal generators, three power amplifiers, and a trio of three‐axial Helmholtz electromagnetic coils (HEC) mounted on an inverted optical microscope (Nikon Eclipse Ti‐U). Signal generators produced electrical signals, amplified by power amplifiers to drive the HEC, resulting in the creation of a highly uniform magnetic field with easily controlled orientation. To offer flexible adjustment of the magnetic field direction, the gamepad was customized with various functions, enabling intuitive manipulation while the signal generators adjust accordingly. Programming was conducted in C++, utilizing the Windows Multimedia Joystick API for controller input. For deeper insight into the system's functioning, please refer to Text S2 (Supporting Information).

### Experimental Observation and Analysis

The propulsion of the BMT was observed under an inverted microscope (Nikon Eclipse Ti‐U, using ×10 and ×20 objectives), equipped with an ultra‐high‐speed camera (Phantom v2512 or TMX 7510) for imaging. The sample solution containing the BMT was positioned in an observation cell within the uniform magnetic field generated by the HEC system. H_2_O_2_ concentration ranged from ≈3 – 5% (v/v), with images captured by the ultra‐high‐speed camera at frame rates up to 450 000 fps. Analysis of experimental movies was conducted using Video Spot Tracker and ImageJ software, with a precision of ≈ ±0.3 µm in particle position determination when using the ×20 objective. Manual contour tracing was employed for frames where surface waves interacted with the BMT, ensuring accurate tracking and mitigating tracking errors caused by refractive index variations.

### Animal Maintenance

All mice were bred and reared in the animal facility of Tsinghua University at 22 °C with a 12‐h light/dark cycle (lighting time 7:00 ‐ 19:00). Food and water are freely available. All animal studies were conducted under the guidance of the Animal Care and Utilization Committee (IACUC) of Tsinghua University. According to the National Institutes of Health “Animal Ethical Use Guidelines”, the experimental procedure has been approved by the Laboratory Animal Care and Use Management Committee of Tsinghua University and the Beijing Municipal Science and Technology Commission (assigned number: SYXK‐2019‐0044).

### Embryo Culture and Collection

Embryos were obtained from Institute of Cancer Research (ICR) female mice at the E3.75–E4.0 stage. Female mice (aged 2–3 months) received intraperitoneal injections of 10 international units (IU) of pregnant mare serum gonadotropin (PMSG) (Solarbio, P9970), followed by a subsequent injection of 10 IU of human chorionic gonadotropin (hCG) after 48 h. After hCG injection, superovulated female mice were paired immediately with male mice aged 3 to 4 months. Pre‐implanted embryos at E3.75 to E4.0 post‐mating were flushed from the uterus using KSOM (Caisson, IVL04) and cultured in droplets of KSOM, covered with pre‐warmed mineral oil (Sigma, M8410), at 37 °C in a 5% CO_2_ atmosphere. Both mineral oil and KSOM were pre‐warmed in the incubator for a minimum of 30 min before embryo culture. Embryo extraction and transfer were performed using homemade glass capillary tubes under a stereo microscope platform (Olympus SZX16).

### Statistical Analysis

Experimental data of mass and density are presented in a way of fitting value ± uncertainty of 90% confidence level. The R‐squared values of fitting in this work are >0.928. *N* = 10 independent measurements using different microparticles were performed to obtain the master curve for H_2_O_2_ solution, and *n* = 5 samples were used to obtained the master curve for embryo measurement in H_2_O_2_ and sucrose solution. Matlab was used for statistical analysis.

## Conflict of Interest

The authors declare no conflict of interest.

## Author Contributions

L.W. and M.S. contributed equally to this work. H.C., X.Z., and L.W. performed conceptualization; L.W., M.S., C‐L. L., H.L., P.N., X.L., Z.G., J.C., X.W., and A.H. performed data curation; L.W., M.S., L.C., F.Y., and X.Z. performed formal analysis; X.Z., H.C., J.D., L.L., D.G., and L.W. performed funding acquisition; L.W., M.S., L.C., F.Y., X.Z., and H.C. performed investigation; L.W., M.S., L.C., F.Y., H.L., P.N., X.L., Z.G., J.C., X.W., and L.L. performed methodology; X.Z. and H.C. performed project administration; X.Z., H.C., J.D., D.G., and L.L. acquired resources; L.C., L.W., and F.Y. acquired software; X.Z., H.C., and J.D. performed supervision; L.W., L.C., X.Z., H.C., and L.L. performed validation; L.W., M.S., L.C., F.Y., H.L., and P.N. performed visualization; L.W., X.Z., and M.S. wrote the original draft; X.Z., L.W., H.C, and L.C. wrote the review and performed editing.

## Supporting information

Supporting Information

Supplemental Video 1

Supplemental Video 2

Supplemental Video 3

## Data Availability

The data that support the findings of this study are available from the corresponding author upon reasonable request.
