# Supplementary material for: Sub‐Nanogram Resolution Measurement of Inertial Mass and Density Using Magnetic‐Field‐Guided Bubble Microthruster
Source: Adv Sci (Weinh). 2024 May 22;11(29):2403867. doi: 10.1002/advs.202403867 (PMC11304303; doi:10.1002/advs.202403867)
Supplement: Supplementary file 1 — Supporting Information [file ADVS-11-2403867-s002.docx]

**Supporting Information of**

**Sub-nanogram Resolution Measurement of Inertial Mass and Density Using Magnetic-field Guided Bubble Micro****thruster**

Leilei Wang,^1#^ Minjia Sheng,^2#^ Li Chen,^2^ Fengchang Yang,^1^ Chenlu Li,^2^ Hangyu Li,^1,3^ Pengcheng Nie,^1,3^ Xinxin Lv,^4^ Zheng Guo,^4^ Jialing Cao,^4^ Xiaohuan Wang,^1^ Long Li,^1^
Anthony L. Hu,^5^ Dongshi Guan,^1,3*^ Jing Du,^4*^ Haihang Cui,^2*^ and Xu Zheng,^1*^

1. State Key Laboratory of Nonlinear Mechanics, Beijing Key Laboratory of Engineered Construction and Mechanobiology, Institute of Mechanics, Chinese Academy of Sciences, Beijing 100190, China

2. School of Building Services Science and Engineering, Xi’an University of Architecture and Technology, Xi’an 710055, China

3. School of Engineering Science, University of Chinese Academy of Sciences, Beijing 100049, China

4. Key Laboratory for Biomechanics and Mechanobiology of Ministry of Education, Beijing Advanced Innovation Center for Biomedical Engineering, School of Biological Science and Medical Engineering, Beihang University, Beijing 100083, China

5. The High School Affiliated to Renmin University of China, Beijing 100080, China

*Corresponding authors:

Xu Zheng: zhengxu@lnm.imech.ac.cn, orcid.org/0000-0002-2398-9283.

Haihang Cui: cuihaihang@xauat.edu.cn.

Jing Du: dujing@buaa.edu.cn.

Dongshi Guan: dsguan@imech.ac.cn, orcid.org/0000-0002-4433-3662.

# L. W., and M. S. contributed equally to this work.

**A list of supplemental videos**:

Video S1. The working principle of inertial mass/density measurement based on the BMT.

Video S2. Different working modes of the BMT: pusher, puller and anchor.

Video S3. The inertial response of the embryo to the impact of the BMT.

## Text S1. Characterizing the Hollow Glass Microsphere (HGM)


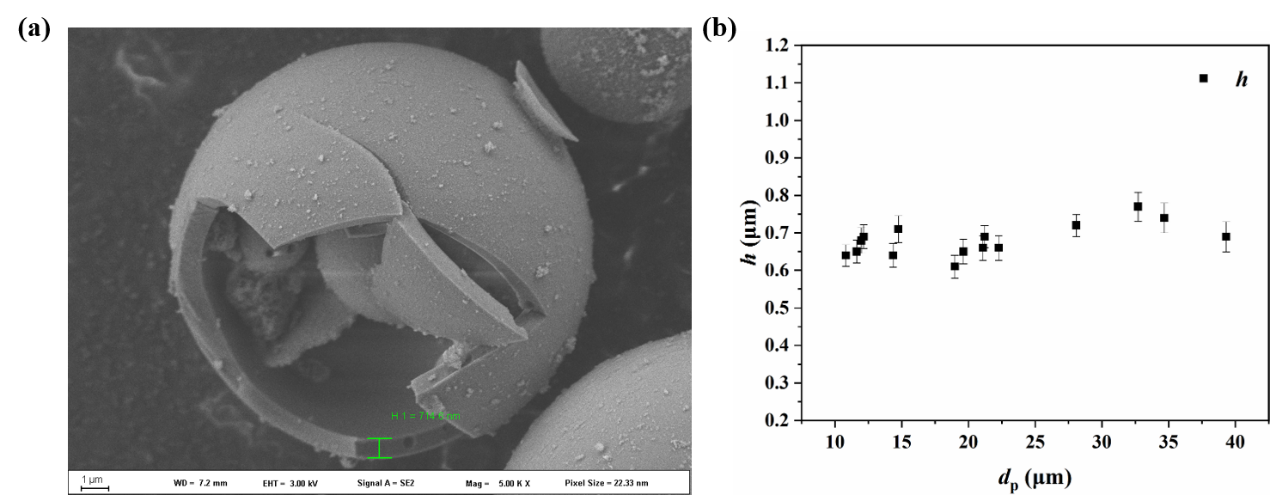


**Figure. S1.** (a) SEM image (in the back scatter mode) and (b) the measured data of the shell thickness *h* for different HGMs with diameter *d*_p_.





**Figure. S2.** Theoretical relation between the effective density and the diameter *d_p_* of the HGM based on **eq. (4)** and **eq. (5)**. Here*, ρ*_HGM_, *ρ*_HGM-Pt-20nm_ and *ρ*_HGM-Pt-40nm_ represents the density HGM with no coating, 20 nm and 40 nm of coated Pt layer on the hemisphere of the HGM, respectively.

**Tabel. S1** Theoretical relation between the effective density and the diameter *d_p_* of the HGM based on **eq. (4)** and **eq. (5)**.

| **Diameter (μm)** | ***ρ*_HGM_ (g/cm^3^)** | ***ρ*_HGM-Pt-20nm_ (g/cm^3^)** | ***ρ*_HGM-Pt-40nm_ (g/cm^3^)** |
| --- | --- | --- | --- |
| 10 | 0.812 | 0.941 | 1.069 |
| 12 | 0.693 | 0.801 | 0.908 |
| 12.8 | 0.655 | 0.756 | 0.856 |
| 14 | 0.605 | 0.697 | 0.789 |
| 16 | 0.536 | 0.617 | 0.697 |
| 18 | 0.481 | 0.553 | 0.624 |
| 20 | 0.437 | 0.501 | 0.566 |
| 20.4 | 0.429 | 0.492 | 0.555 |
| 22 | 0.400 | 0.458 | 0.517 |
| 24 | 0.369 | 0.422 | 0.476 |
| 26 | 0.342 | 0.391 | 0.441 |
| 28 | 0.319 | 0.365 | 0.411 |
| 30 | 0.298 | 0.341 | 0.384 |
| 31.6 | 0.284 | 0.325 | 0.366 |
| 32 | 0.281 | 0.321 | 0.361 |
| 34 | 0.265 | 0.303 | 0.341 |
| 36 | 0.251 | 0.287 | 0.322 |
| 38 | 0.238 | 0.272 | 0.306 |
| 40 | 0.227 | 0.259 | 0.291 |

**Test S2. The scaling law of *γ* vs. *δ***

Based on the law of conservation of momentum, given the energy of the bubble growth and collapse is evenly distributed and transferred to the JM and the microparticle, the following equation can be obtained：

$\frac{4}{3}\pi{R_{P}}^{3}\rho_{p}\bar{Vp}=\frac{4}{3}\pi{R_{JM}}^{3}\rho_{JM}\bar{V_{JM}}$ S(1)

where *R_p_*, *ρ_p_*, *R_JM_*, *ρ_JM_* denote the radii and densities and of microparticle and JM respectively. $\bar{V_{p}}$ denotes the average velocity of the microparticle in a bubble cycle, while $\bar{V_{JM}}$ denotes the average velocity of the JM without loading the microparticle. Here, we define *γ* = *R_p_*/*R*_JM_, and *δ* = $\bar{V_{p}}$/$\bar{V_{JM}}$. The scaling of *γ* ~*δ*^-1/3^ can be obtained by simplifying **eq. S(1)**.

**Text S3**. **Numerical simulation.**

In the presented study, the computational strategy employed encompasses the Finite Volume Method (FVM) for the resolution of the Navier-Stokes equations, pivotal in the analysis of fluid dynamics. Central to our investigation is the interface dynamics between bubbles and the free surface, for which the Volume of Fluid (VOF) approach is employed due to its extensive validation in tracking bubble and free surface interfaces. The core equations governing this model are expressed as follows,

$\frac{\partial\rho}{\partial t}+\nabla\cdot(\rho U)=0$ S(2)

S(3)

where, $\sigma$is the surface tension coefficient, $\kappa$is the surface curvature, and $\sigma\kappa\hat{n}$is the force term of surface tension. The Continuum Surface Force (CSF) model is instrumental in encapsulating the effects of surface tension within this framework. In the realm of the VOF methodology, the volume fraction *α*_q_ of a given phase Q adheres to a scalar transport equation, signifying the conservation of mass across the interface,

$\frac{\partial\alpha_{q}}{\partial t}+\left(U\cdot\nabla\right)\alpha_{q}=0$ S(4)

The initial parameters for the simulation specify that the inception size of the bubble matches its maximum diameter during the growth phase, with the velocity at the bubble-liquid interface approximated to zero. The internal pressure of the bubble is set to the saturated vapor pressure (3540 Pa), a crucial consideration given that the collapse of the bubble induces localized zones of elevated pressure, velocity, and temperature within the fluid. This necessitates the incorporation of the equation of state for the liquid to accurately model these effects,

$p=\frac{\rho_{0}c_{0}^{2}}{n_{l}}\left(\left(\frac{\rho}{\rho_{0}}\right)^{n_{l}}-1\right)+p_{0}$ S(5)

Furthermore, the gas phase is governed by its own equation of state,

$p=\rho R_{g}T$ S(6)

and the energy dynamics within the system are captured by the energy equation,

$\frac{\partial}{\partial t}\left(\rho E\right)+\nabla\cdot\left[U\left(\rho E+p\right)\right]=\nabla\cdot\left(k_{eff}\nabla T\right)$ S(7)

To account for the effects of rigid body motion on the flow field, a dynamic mesh model is implemented. This approach facilitates the computation of forces acting on particles, which, through the application of Newton's second law, enables the calculation of particle velocity and displacement. Subsequent adjustments to boundary positions necessitate the local deformation or regeneration of the mesh.

In the specific context of simulating the three-dimensional behavior of the BMT, the computational domain is delineated in Figure. S3, with dimensions (50x20x20) *R*_JM_. A bubble at its peak growth stage diameter is positioned in proximity to the gas-liquid interface. The symmetry axis of the bubble and microparticle is co-linearly arranged. The JM is slightly lower than that of the microparticle, according to the experimental observation. The diameters of bubble, particle and JM are consistent with the experiment. The grid independence test validates a minimum grid length of 0.1 RJM for the interface evolution region, and the temporal resolution for transient analyses is set at a minimum timestep of 10^–9^ s. This discretization strategy ensures second-order spatial accuracy across the control equations, underpinning the fidelity of the simulation outcomes.


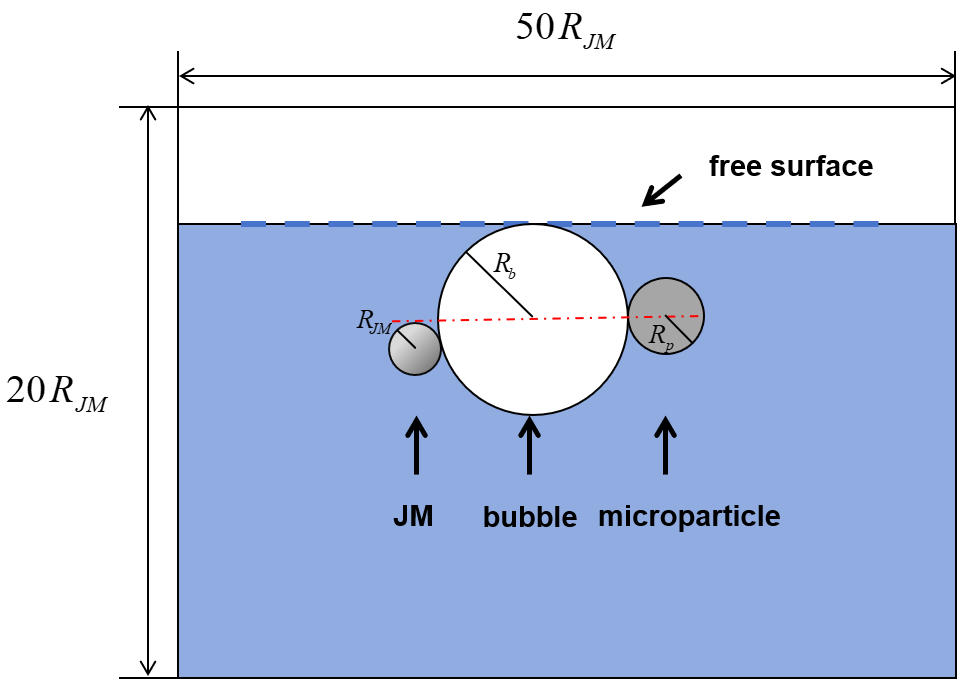


**Figure. S3.** Computational domain settings for bubble collapsing and pushing.

**Text S4**. **Magnetic manipulation system.**

The magnetic manipulation system consists of hard ware and software. The details are described below.

**Hardware**

The magnetic manipulation system's hardware comprises a three-dimensional Helmholtz electromagnetic coil (3D-HEC) with 1000 turns per coil, two dual-channel signal generators (ATF20F), three power amplifiers (ATA-309), an inverted microscope (Nikon Eclipse-Ti), two objective lenses (10x with NA = 0.3 and 20x with NA = 0.45), a high-speed CCD camera (Phantom V2012/ TMX7510), and a gamepad. The uniform magnetic field generated by the HEC can be adjusted in direction and intensity by manipulating the voltage from the signal generator, which is then amplified by the power amplifier. For magnetic modulation, we programmed various functions into the gamepad, enabling immediate manual adjustments to the magnetic field. The locomotion and various working modes of the system are captured by the high-speed CCD camera mounted on the inverted microscope.

**Software**

To achieve flexible and real-time control of the magnetic field, we programmed various functions for the gamepad to manipulate the signal generator. The programming language chosen is C++, and the interface for joystick control is the Windows Multimedia Joystick API. Additionally, the serial port programming, adhering to the RS-232 standard interface, relies on the System. IO. Ports namespace functionality provided by the .NET Framework 4.0.
